# Impact of Co-Occurring Psychiatric Disorders on Retention in a Methadone Maintenance Program: An 18-Month Follow-Up Study

**DOI:** 10.3390/ijerph6112822

**Published:** 2009-11-12

**Authors:** Mònica Astals, Laura Díaz, Antònia Domingo-Salvany, Rocío Martín-Santos, Antoni Bulbena, Marta Torrens

**Affiliations:** 1 Institute of Psychiatry and Addiction (IAPs), Hospital del Mar and Institut Municipal d’Investigació Mèdica (IMIM), Hospital del Mar, Psg. Marítim 25-29, E-08003 Barcelona, Spain; E-Mails: mastals@imas.imim.es (M.A.); ldiaz@imim.es (L.D.); rmsantos@imim.es (R.M.S.); 16359@imas.imim.es (A.B.); 2 Research Programme in Epidemiology and Public Health, Institut Municipal d’Investigació Medica (IMIM), Hospital del Mar, Doctor Aiguader 88, E-08003 Barcelona, Spain; E-Mail: adomingo@imim.es; 3 Department of Psychiatry, Universitat Autònoma de Barcelona, Barcelona, Spain

**Keywords:** methadone, retention, co-occurring psychiatric disorders, incidence

## Abstract

We assess the influence of co-occurring psychiatric disorders on retention in 189 opioid dependent patients in a methadone maintenance treatment (MMT) and determine the incidence of psychiatric co-morbidity during an 18-month follow-up period. About 68.5 % were retained in the MMT. Neither co-occurring mental disorders (chi-square = 0.303, df = 1, p = 0.622) nor methadone doses [85 (88.9) *vs.* 79.2 (85) mg/day, p = 0.672] were related to retention. In the follow-up period 19 new diagnoses were made, mainly major depression and antisocial and borderline personality disorders. Co-occurring psychiatric disorders should be assessed during MMT follow-up.

## 1. Introduction

In the management of heroin dependent subjects methadone maintenance treatment (MMT) programs are effective in reducing heroin use, crime related to drug use, HIV risk behaviors, overdose mortality [1–3] and improving quality of life [4,5]. Effectiveness is related to the effect of methadone on retaining patients in the program. Research into the factors related to retention in MMT is an important issue in order to improve the effectiveness of MMT.

Previous studies suggest that the patient must stay for at least one year in the MMT for the treatment to be effective [6,7] although longer treatment is recommended. In different studies, the 1-year retention rate ranges between 25% to 82% [6,8–13]. Factors associated with retention rate are methadone dosage [2,14], differences between treatment settings [15], severity of drug use at the time of enrolment in the program [16] and age at time of MMT entrance [17]. The influence of co-occurring mental disorders other than substance abuse has also been studied, although results remain controversial [11,13,15,18]. This could be mainly related to the difficulties in diagnosing co-occurring disorders in substance users [19,20] and to the fact that in previous studies the patients were only assessed at the time of admission into MMT and therefore the role of new non-substance use psychiatric diagnosis during the follow-up period was not considered.

A prospective follow-up study of opioid dependent patients included in the MMT [21] was carried out with the following objectives: (1) to assess the influence of co-occurring disorders on retention in the MMT program and (2) to determine the incidence of co-occurring disorders during an 18-month follow-up.

## 2. Methods

### 2.1. Sample

The study participants were 189 opioid dependent patients (77% male, mean age 34 ± 7.5 years) consecutively admitted to a MMT in Barcelona, Spain, and followed up to 18 months.

### 2.2. Procedure

In the baseline visit, after full explanation of the purposes of the study, written informed consent was obtained and patients were assessed with the PRISM-IV and a close-ended questionnaire (see other variables). At follow-up (18 months) patients were reassessed with the same measures. The study protocol was approved by the institutional review board.

### 2.3. Methadone Maintenance Treatment

The MMT provided at the Drug Abuse Out-patient Centre (CAS-BARCELONETA) is a low-threshold MMT, that is to say such program is not abstinence oriented. The only requirement for inclusion in the program is a definitive diagnosis of opioid dependence according to DSM-IV criteria. Forced discharge only occurs for aggressive behavior and drug trafficking in the centre. There is a high dose policy (no upper limit) and no restriction on long-term treatment (no time limit). The induction period on MMT lasts about 1–2 months until the stable maintenance doses is achieved. Urine toxicology screens are carried out randomly once a week under supervision. Methadone is dispensed to patients daily on the form of syrup with orange juice and has to be ingested in the presence of a nurse. Take-home methadone doses are provided when weekly urine screening tests are repeatedly clean (at least for a period of one month). Take-home privileges are revoked in response to positive urine tests results, and patients are referred to the clinicians to assess a possible increase in methadone doses. In addition to methadone and urine drug screen, individual counseling is the major therapeutic vehicle and frequency varies depending upon the stage of treatment and patient needs. Counseling focused on encouraging reduced drug use and helping patients to cope with problems (either through direct counseling or referral to other services) that made them more vulnerable to continued drug use is provided.

### 2.4. Current Co-Occurring Diagnoses

Diagnoses of current substance use disorder (SUD) and co-occurring mental disorders, were carried out according to DSM-IV criteria and using the Spanish version of the Psychiatric Research Interview for Substance and Mental Disorders (PRISM-IV) [22], administered by two trained psychologist researchers with clinical experience with patients with substance abuse or mental disorders. The PRISM-IV has shown a good test-retest reliability [23] and validity [22] in substance abusers.

### 2.5. Other Variables

Baseline patient’s sociodemographic characteristics (including employment and legal status), and drug use and sexual risk behaviors, substance use variables, and infection by HIV and hepatitis C virus were collected with a close-ended questionnaire [21]. At follow-up, the questionnaire included data on MMT provided (methadone dose received at 18 months). In the patients not retained in the program, data on the last methadone dose administered before interruption of MMT was used.

### 2.6. Statistical Analysis

All statistical analyses were carried out with the SPSS statistical software package [SPSS V14; Chicago, IL, USA]. Retention in the MMT was defined as remaining in the same MMT program, and after stable doses of methadone was reached. The presence of co-occurring mental disorders at baseline between retained and non-retained patients was compared with Pearson’s chi-square test. Fisher’s exact test was used when one or more cells of the contingency tables had expected counts of less than five.

To determine which variables were associated with MMT retention, we compared retained versus non-retained patients in the following baseline variables: sociodemographic (sex, age, education level, employment, legal status); psychopathological (co-occurring mental disorders [yes/no]); use of the following substances during the last month: alcohol, sedatives, cocaine, cannabis, opiates other than heroine; SUD diagnoses (yes/no) of the following substances: alcohol, sedatives, cocaine, cannabis, opiates other than heroine; VIH/Hepatitis C risk-related variables: i.v. route use, sharing injection material, sexual risk behavior (yes/no for always using condoms); presence or absence of HIV-Ab and/or HCV-Ab; and methadone dose. Comparisons were made by means of Pearson’s chi-square test or Fisher’s exact test (for categorical variables) and Student’s *t*-test (for continuous variables). A logistic regression analysis with retention as the dependent variable and those of the former variables that showed a *p*-value of less than 0.25 in the univariate analyses: sex, age, educational level, and current diagnosis of cocaine and opiates other than heroin dependence as independent variables was done. The goodness-of-fit of the model was assessed by the Hosmer–Lemeshow test.

To assess the incidence of co-occurring disorders during the follow-up, the cumulative incidence (ratio of incident cases divided by those subjects without co-occurring disorders at baseline) and the incidence rate (ratio of new diagnoses divided by total time of follow-up) of retained cases were calculated.

Exact 95 % confidence intervals for the incidence rate based on the Poisson distribution were calculated.

## 3. Results

Table 1 shows a summary of the baseline characteristics of the sample differentiating among those 128 (67.7%) cases with only a current SUD diagnosis and 61 (32.3%) with a current co-occuring mental disorders. Anxiety disorders were the most frequent Axis-I diagnoses, followed by mood disorders, and psychotic disorders. More than 20% of the sample fulfilled the criteria for antisocial or borderline personality disorder.

**Figure 1.**
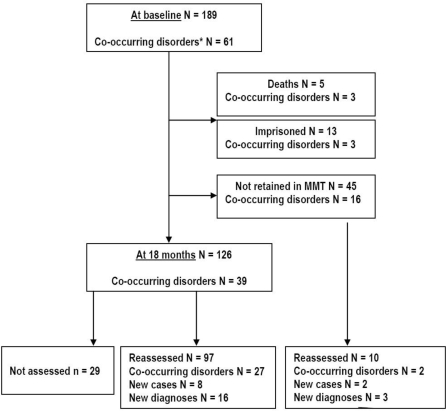
Situation of the 189 opioid dependent patients admitted to a MMT at 18 months follow-up. * Co-occurring disorders: co-occurring substance use [abuse or dependence] and mental disorders.

The situation of patients during the 18-month follow-up period is shown in Figure 1. Sixty-three of the 189 patients dropped out the study because of death, imprisonment or MMT discontinuation. Therefore, a total of 126 patients were followed, with a retention rate of 68.5%. Some baseline differences between the 126 patients who continued in the MMT and the 63 patients who dropped out were found. Subjects that dropped out used more cocaine, (57.1% *vs.* 38.1%; chi square = 7.510, df = 2, p = 0.023), reported more i.v. route (65.1% *vs.* 42.9%; chi square = 8.297, dfl = 1, p = 0.005) and less sexual risk behaviors (36.5% *vs.* 63.5%; chi square = 12.333, dfl = 1, p = 0.001) during last 30 days before starting in the MMT. Furthermore, subjects that dropped out had more past (68.3% *vs.* 51.6%; chi square = 4.764, df = 1, p = 0.030) and current (44.4% *vs.* 27%; chi-square = 5.809, df = 1, p = 0.021) cocaine dependence diagnoses than subjects who continued in the MMT.

Twenty-nine patients were not available for reassessment at follow-up, even if still in MMT. Baseline differences were found respect to those 97 patients retained in MMT and reassessed at follow up. The 29 who did not accepted to be reassessed used more the i.v. route (65.5% *vs.* 36.1%; chi square = 7.898, df = 1, p = 0.006) and higher alcohol (17.2% *vs.* 13.4%; chi square = 7.236, df = 2, p = 0.027) and cocaine (58.6% *vs.* 19.6%; chi square = 17.731 df = 2, p < 0.001) in the 30 days before entering MMT. Furthermore, the 29 patients had more antisocial personality disorder diagnoses (17.2% *vs.* 4.1%; chi square = 5.792, df = 1, p < 0.030) (data not shown).

Of the 61 patients with co-occurring mental disorders at baseline, 39 (63.9 %) were retained in the MMT programme and 22 (36.1 %) were lost to follow-up, whereas from the 128 without co-occurring mental disorders, 87 (68%) patients were retained (chi-square = 0.303, df = 1, p = 0.622). No difference in baseline characteristics were observed between the patients retained in MMT and available for the reassessment at time of follow-up (n = 97) and those retained but not available for the reassessment (n = 29). The difference between methadone dosage of those patients retained in the MMT and last dose administered in those who dropped out was not statistically significant [85.5 (88.9) *vs.* 79.2 (85) mg/day, p = 0.672]. None of the variables included in the multivariate model were significant predictors of treatment retention, although being male (OR: 2.59, 95% CI: 0.98–6.84; p = 0.055) and lower educational level (OR: 2.65, 96% CI: 0.98–7.13; p = 0.054) were nearly significant predictors of non-retention. The p-value of the Hosmer-Lemeshow tests was 0.47 indicating that the model fit was satisfactory.

A total of 107 patients were available for the assessment of co-occurring mental disorders at follow-up. Co-occurring disorders were established in 29 subjects. When patients with (n = 29) and without (n = 78) co-occurring disorders were compared, those with co-occurring disorders had worked fewer months before admission in the MMT (41.4% *vs.* 69.2%, p = 0.013) and before reassessment at follow-up (24.1% *vs.* 50%, p = 0.027), showed higher percentage of current alcohol abuse diagnoses (13.8% *vs.* 1.3%, p = 0.019) and had received more psychiatric treatment (44.8% *vs.* 19.2%, p = 0.012).

In 10 of the 107 patients, at least one new co-occurring disorder was diagnosed, with a total of 19 diagnoses. Sixty percent of these 10 patients were men, with a mean age of 31.60 (5.73) years. The cumulative incidence of co-occurring disorders was 13% (95% CI 6.4% to 22.5%). The cumulative incidence in the 97 patients retained in the MMT program and reassessed was 11.43 % (95% CI 5.07% to 21.28%) compared with 28.6% (95% CI 3.7% to 71%) in 10 patients reassessed but not retained in the MMT program.

Of the 19 new diagnoses, 16 were made in the 97 patients retained in MMT and reassessed, and 3 in the subsample of 10 patients not retained but reassessed. The incidence rate of co-occurring disorders at follow-up was 0.11 diagnoses per year (95% CI 0.06 to 0.18) in patients retained in MMT and 0.20 (95% CI 0.04 to 0.58) in those not retained. Nine new Axis I diagnoses were done: major depression (five diagnoses), anxiety disorders (one panic disorder and one specific phobia) and psychotic disorder (one induced and one primary). Regarding Axis II disorders, 10 new diagnoses had been made of personality disorders (five antisocial personality disorder [APD] and five borderline personality disorder [BPD]); see Table 2 for more details.

## 4. Discussion

In this study, the overall cumulative incidence of co-occurring disorders in methadone-treated opioid dependent patients followed-up for 18 months was 13%. Major depression and antisocial and borderline personality disorders were the most common new diagnoses. The cumulative incidence, although not statistically significant, was lower in patients retained in MMT than those not retained. Moreover, co-occurring disorders at the time of inclusion in the MMT was not related to retention in treatment.

Major depression has been the main incident Axis I diagnoses. Interestingly, all those patients had been diagnosed with an anxiety disorder at baseline. Our results are similar to those obtained by others at 12 months follow-up after discharge of inpatient detoxification treatment using similar methodology [20], but differ from those of Grant *et al.* [24], suggesting that anxiety disorders at baseline more often predicted incidence of anxiety disorders rather than mood disorders in the general population. The establishment of new personality disorders was indeed quite surprising, since a personality disorder implies a pattern of behaviors developing through one’s adult life, beginning in the early adulthood. Such result could be explained by the fact that at baseline patients were diagnosed just at admission of MMT and it was more difficult to differentiate personality than substance related symptoms. In agreement with other authors [13,25], we consider that the diagnoses of personality disorders should be done once the patients have become clinically stabilized.

Although in the univariate analysis, cocaine use as IV use at entrance, was a predictor of drop-out of treatment in agreement with previous studies[26], in the multivariate regression analysis, no significant relationship was proven between these variables and the retention in MMT. Nor did we find other significant predictor of retention in MMT, although statistical significance was almost reached for two variables (gender and education level). It seems that patients with a lower educational level might need some specific treatment approaches, such as mapping-enhanced counseling to improve treatment outcomes. Previous studies [27,28] found controversial results regarding education level and MMT retention. We did not observe a relationship between methadone dose and retention in treatment, probably because mean dose of methadone administered (near 80 mg/day in both groups) was in the recommended range [14]. Psychiatric comorbidity did not show any influence on patient’s retention in MMT, as previously described by other authors[11,13,18].

Some study limitations should be mentioned. Firstly, the lack of the assessment of psychosocial interventions received during the treatment, and secondly, the fact that co-occurring mental disorders were only evaluated in a small percentage of subjects not retained in the MMT.

In summary, the present study shows a relatively high cumulative incidence of co-occurring diagnoses (mainly major depression) among opioid dependent patients available at follow-up. Furthermore, the unexpected rate of new personality disorder diagnoses established in MMT retained patients enhances the relevance of careful detection of co-occurring mental disorders not only at the time admission to the treatment, but also throughout the whole program.

## Funding and Support

Conflicts of interest: Not declared. This study was supported by grants from “Plan Nacional sobre Drogas” (2SI/01/07) and “Fondo de Investigacion Sanitaria” (FIS) (G03/005 and PI052086), Madrid, Spain.

## Figures and Tables

**Table 1.** Differences between patients with/without co-occurring disorders at baseline.

**Variable**	**Co-occurring disorders N = 61 (%)**	**Only SUD N = 128 (%)**	**χ^2^ or *t***	**df**	***p***
Women	20 (32.8)	24 (18.8)	χ^2^ = 4.557	1	**0.043**

Age, years, mean (SD)	33.34 (8.8)	34.1(6.8)	*t =* −0.637	187	0.525

Marital status					
Single	42 (68.9)	60 (46.9)			
Currently married	12 (19.7)	45 (35.2)	χ^2^ = 8.079	2	**0.018**
Separated/divorced	7 (11.5)	23 (18)			

Education level					
Primary School	29 (47.5)	47 (36.7)			
Secondary School	20 (32.8)	50 (39.1)	χ^2^ = 2.018	2	0.365
University	12 (19.7)	31 (24.2)			

Any month employed last six months	26 (42.6)	87 (68)	χ^2^ = 11.039	1	**0.001**

Legal status					
Any detention last six months	20 (32.8)	24 (18.8)	χ^2^ = 4.557	1	**0.043**
Any imprisonment last 6 months	7 (11.5)	11 (8.6)	χ^2^ = 0.398	1	0.598

Serological status					
HIV—Ab positive	18 (29.5)	27 (21.8)	χ^2^ = 1.329	1	0.276
HCV—Ab positive	39 (63.9)	58 (48.7)	χ^2^ = 3.747	1	0.059

HIV current risk behaviors last six monthsMonths	38 (62.3)	57 (44.5)	χ^2^ = 5.215	1	**0.029**
Any i.v. drug use	30 (49.2)	73 (57)	χ^2^ = 1.027	1	0.350
Any sexual risk behavior					

Drug use last 30 days					
Alcohol	5 (8.2)	23 (18)	χ^2^ = 3.127	2	0.209
Cocaine	10 (16.4)	15 (11.7)	χ^2^ = 2.402	2	0.301
Sedatives	15 (24.6)	16 (12.5)	χ^2^ = 4.423	2	0.110
Other opiates	9 (14.8)	21 (16.4)	χ^2^ = 0.084	2	0.834
Cannabis	18 (29.5)	35 (27.3)	χ^2^ = 0.586	2	0.746

Cocaine route use					
Snorted/Smoked	13 (38.2)	27 (50)	χ^2^ = 1.165	1	0.380
Injected	21 (61.8)	27 (50)			

Heroine route use					
Smoked/Inhaled	12 (18.7)	37 (28.9)			
Snorted	11 (18)	33 (25.8)	χ^2^ = 4.770	1	0.092
Injected	38 (62.3)	58 (45.3)			

Abuse or dependence diagnoses					
Alcohol	6 (9.8)	12 (9.4)	χ^2^ = 0.010	1	1
Other opiates	4 (6.6)	9 (7)	χ^2^ = 0.014	1	1
Cocaine	25 (41)	43 (33.6)	χ^2^ = 0.979	1	0.335
Sedatives	11 (18)	18 (14.1)	χ^2^ = 0.501	1	0.520
Cannabis	17 (27.9)	20 (15.6)	χ^2^ = 3.934	1	0.053
Stimulants	2 (3.3)	2 (1.6)	χ^2^ = 0.587	1	0.596

Non-SUD co-occurring diagnoses					
Only Axis I	32 (52.5)	-			
Only Axis II	22 (36.1)	-			
Both Axis I + II	7 (11.5)	-			

Major depression	9 (14.8)				
Induced depression	5 (8.2)				
Schizophrenia	5 (8.2)				
Panic w/without agoraphobia	12 (19.7)				
Social phobia	10 (16.4)				
Simple phobia	8 (13.1)				
Post traumatic stress	-				
Obsessive compulsive	4 (6.6)				
Bulimia	1 (1.6)				

Antisocial Personality Disorder	16 (26.2)				
Borderline Personality Disorder	13 (21.3)				

Co-occurring diagnoses: co-occurring substance use [abuse or dependence] and mental disorders; Only SUD: Only substance use disorders.

**Table 2.** Co-occurring disorders incidence in a cohort of 107 patients reassessed at 18 months.

	**Co-occurring disorders**			
**Disorders at baseline**	**Baseline**	**Follow-up**		
		**Axis I**	**Axis II**	**Axis I & II**
**Only SUD**	77	1 Induced Psychosis^a^ 1 Simple Phobia 1 Schizophrenia	1 APD & BPD^a^,^b^ 1 APD 2 BPD	
**Non SUD co-occurring diagnoses**	30			
**Axis I**	19			
Major Depression	7		1 BPD	1 Induced Depression & BPD^b^
Induced Depression	1			
Schizophrenia	2			
Induced Psychosis	-			
Panic w/without Agoraphobia	9	3 Major Depression		
Social Phobia	6		2 APD	
Simple Phobia	4			
Obsessive Compulsive	3		1 APD	
**Axis II**	8			
Antisocial Personality	5			
Borderline Personality	6			
**Axis I & II**	3			
Social Phobia, Induced Depression & BPD	1	1 Major Depression		
Panic with/without Agoraphobia & BPD	1	1 Major Depression		
Panic with/without Agoraphobia & APD	1			

a Patients from the subsample of 10 patients not retained but assessed;

b Both diagnoses in single patient; APD: Antisocial personality disorder; BPD: Borderline personality disorder.
